# Joint analyses of human milk fatty acids, phospholipids, and choline in association with cognition and temperament traits during the first 6 months of life

**DOI:** 10.3389/fnut.2022.919769

**Published:** 2022-08-24

**Authors:** Tengfei Li, Tinu M. Samuel, Ziliang Zhu, Brittany Howell, Seoyoon Cho, Kristine Baluyot, Heather Hazlett, Jed T. Elison, Di Wu, Jonas Hauser, Norbert Sprenger, Hongtu Zhu, Weili Lin

**Affiliations:** ^1^Department of Radiology, University of North Carolina at Chapel Hill, Chapel Hill, NC, United States; ^2^Biomedical Research Imaging Center, University of North Carolina at Chapel Hill, Chapel Hill, NC, United States; ^3^Nestlé Product Technology Center-Nutrition, Société des Produits Nestlé S.A., Vevey, Switzerland; ^4^Department of Biostatistics, University of North Carolina at Chapel Hill, Chapel Hill, NC, United States; ^5^Department of Human Development and Family Science, Fralin Biomedical Research Institute at Virginia Tech Carilion, Virginia Polytechnic Institute and State University, Roanoke, VA, United States; ^6^Department of Psychiatry, University of North Carolina at Chapel Hill, Chapel Hill, NC, United States; ^7^Institute of Child Development, University of Minnesota, Minneapolis, MN, United States; ^8^Division of Oral and Craniofacial Health Science, Adams School of Dentistry, University of North Carolina at Chapel Hill, Chapel Hill, NC, United States; ^9^Nestlé Institute of Health Sciences, Société des Produits Nestlé SA, Lausanne, Switzerland

**Keywords:** human milk, cognition and temperament, Mullen Scales of Early Learning, IBQ, receptive language, surgency, ARA, DHA

## Abstract

Early dietary exposure *via* human milk nutrients offers a window of opportunity to support cognitive and temperament development. While several studies have focused on associations of few pre-selected human milk nutrients with cognition and temperament, it is highly plausible that human milk nutrients synergistically and jointly support cognitive and behavioral development in early life. We aimed to discern the combined associations of three major classes of human milk nutrients with cognition and temperament during the first 6 months of life when human milk is the primary source of an infant’s nutrition and explore whether there were persistent effects up to 18 months old. The Mullen Scales of Early Learning and Infant Behavior Questionnaires—Revised were used to assess cognition and temperament, respectively, of 54 exclusively/predominantly breastfed infants in the first 6 months of life, whose follow-ups were conducted at 6–9, 9–12, and 12–18 months old. Human milk samples were obtained from the mothers of the participants at less than 6 months of age and analyzed for fatty acids [total monounsaturated fatty acids, polyunsaturated fatty acid, total saturated fatty acid (TSFA), arachidonic acid (ARA), docosahexaenoic acid (DHA), ARA/DHA, omega-6/omega-3 polyunsaturated fatty acids ratio (n-6/n-3)], phospholipids [phosphatidylcholine, phosphatidylethanolamine (PE), phosphatidylinositol (PI), sphingomyelin], and choline [free choline, phosphocholine (PCho), glycerophosphocholine]. Feature selection was performed to select nutrients associated with cognition and temperament. The combined effects of selected nutrients were analyzed using multiple regression. A positive association between the arachidonic acid (ARA) and surgency was observed (*p* = 0.024). A significant effect of DHA, n-6/n-3, PE, and TSFA concentrations on receptive language (*R*^2^ = 0.39, *p* = 0.025) and the elevated ARA, PCho, and PI with increased surgency (*R*^2^ = 0.43, *p* = 0.003) was identified, suggesting that DHA and ARA may have distinct roles for temperament and language functions. Furthermore, the exploratory association analyses suggest that the effects of human milk nutrients on R.L. and surgency may persist beyond the first 6 months of life, particularly surgency at 12–18 months (*p* = 0.002). Our study highlighted that various human milk nutrients work together to support the development of cognition and temperament traits during early infancy.

## Introduction

Human milk has been considered the best and primary source of nutrients for infants, particularly during the first 6 months of life. Human milk contains a wide variety of nutrients, bioactive components, immunological compounds, and commensal bacteria, which are vital for infants’ survival, health, immunity, brain maturation, and cognitive and temperament development (1, 2). Associations between breastfeeding (BF) and improved child cognitive functions have been widely documented (3); exclusive BF promotes an infant’s receptive language (4) and executive function (5) later in life. BF also has a significant effect on temperament development. Children who were exclusively breastfed had higher surgency and regulation, a trait reflecting an inclination toward high levels of positive affectivity (6) and lower negative affectivity than those who were formula-fed (7). BF duration might be negatively associated with infant fussiness and positively associated with infant unpredictability (8) although these influences on the infant may be mediated by maternal sensitivity (9).

Preclinical and human studies thus far have largely focused on three main classes of nutrients in human milk, namely, the fatty acids, phospholipids, and choline/choline metabolites, separately regarding their effects on early cognitive development. Two long-chain polyunsaturated fatty acids (LCPUFAs), namely, docosahexaenoic acid (DHA) and arachidonic acid (ARA), are believed to be involved in infant brain structural and cognitive development (10–13). It was reported that DHA and ARA supplementation is vital for early cognitive development (14–17) and can improve memory and problem-solving scores of preterm infants (18). In addition, Carlson et al. showed that plasma ARA status was strongly correlated with weights and lengths of preterm infants in the first 12 months of life, suggesting that dietary ARA could lead to an improvement in growth in preterm infants (19). In contrast, the potential effects of total saturated fatty acid (TSFA) are controversial. Some argued that increased saturated fat intake was associated with an impaired ability to maintain multiple task sets in working memory and to flexibly modulate cognitive operations, particularly when faced with greater cognitive challenges (20). Phospholipids have also long been shown to be positively associated with brain cognitive processes (21). As major components of biological membranes and particularly abundant in the nervous system (22), phospholipids could act on the hypothalamic–pituitary–adrenal axis (21) and the microbiota–gut–brain axis (23) to exert their beneficial effects on the brain. Choline provides substrates for phosphatidylcholine and sphingomyelin formation, which are essential for neuronal and other cellular membranes, potentially improving signal transduction and brain development (24). Regarding how aforementioned human milk nutrients may be associated with temperament traits, few significant findings have been reported. For example, the total omega-3 LCPUFA was reported to be associated with infant negative affectivity, but this was not significant at an individual level for DHA, eicosapentaenoic acid (EPA), or eicosatetraenoic acid (ETA), three components of the omega-3 LCPUFA (25). Endocannabinoids, a class of ARA derivatives, were shown to be critically important for motivational processes, emotion, stress responses, pain, and energy balance (26).

Despite the well-recognized importance of human milk on infant health, most of the above studies focused only on the effects of an individual human milk nutrient or a class of nutrients. Nutrients in human milk form a biological system (27); single nutrient supplementation is usually overly simplistic, ignoring the existence of other nutrients and their interplays within such a system. While a joint analysis of the micronutrient effects such as vitamins and iron was performed decades ago (28), this has rarely been investigated for the human milk nutrients described above. To this end, we aimed first to examine the combined effects of three major classes of human milk nutrients, namely, fatty acids, phospholipids, and choline, rather than separately evaluating their individual associations with cognition and temperament traits during the first 6 months of life and second to determine whether the effects persist up to 18 months of age.

## Materials and methods

A subset of infants (*n* = 54) enrolled in the Baby Connectome Project—Enriched (BCP-E) study (29) who were exclusively/predominantly breastfed [fed less than four teaspoons or 20 g per day of non-formula and complementary foods/liquids (water, apple juice, etc.)] and younger than 6 months (chronological mean age: 4.43 ± 0.83 months) were included. The rationale for only including exclusively/predominantly breastfed infants was to minimize nutritional contributions from sources other than human milk, i.e., cow’s milk or formula. Children were returned for follow-up visits up to 18 months of age.

Subject recruitment and data collection were conducted by two institutions (University of North Carolina at Chapel Hill, Chapel Hill, NC, and University of Minnesota, Twin Cities, MN). All study activities were approved by the institutional review boards of the two universities. Informed consent was obtained from parents prior to enrolling in the study. The inclusion criteria were as follows: birth at gestational age 37–42 weeks; birth weight appropriate for gestational age; and absence of major pregnancy and delivery complications. The exclusion criteria were as follows: adopted infant; birth weight < 2,000 g; abnormal MR in previous MR imaging; contraindication for MRI; neonatal hypoxia (10-min Apgar score < 5); chromosomal or major congenital abnormality; illness requiring NICU stay > 2 days; significant medical illness or developmental delay, or significant medical and/or genetic conditions affecting growth, development, or cognition (including visual/hearing impairment); the presence of a first-degree relative with autism, intellectual disability, schizophrenia, or bipolar disorder; or maternal preeclampsia, placental abruption, HIV status, and alcohol or illicit drug use during pregnancy.

We used R version 3.6.3 for all the following statistical analyses. We fixed a significance level α = 0.05 and corrected for regression model significance with false discovery rate (FDR) adjustment.

### Human milk collection and macronutrient analyses

Human milk samples were collected at each visit from the second feed of the day whenever possible and from the right breast using a hospital-grade, electric Medela Symphony breast pump. Mothers were instructed to completely express the contents of their right breast to ensure that the collected human milk was representative of nutrients received by the infant across a full feed. The collected human milk samples were vortexed at a maximum speed for 2 min, whose volume and weight were measured and recorded with special attention to avoid bubbles. Subsequently, 3 ml of human milk was used for mid-infrared spectroscopic analyses using the MIRIS Human Milk Analyzer. This step was to ensure that the total fat content (as an indicator of the quality of milk sampling) fell within the expected range. Finally, an aliquot of the minimum 30 ml of volume was transferred from the collection bottle to a 50-ml glass beaker. Using a repeating pipette and an appropriate tip, 11 aliquots of 1 ml of volume were made in 1-ml Eppendorf tubes and nine aliquots of 2 ml of volume were made in 2 ml Eppendorf tubes for storage in a –80°C freezer within 30 min from the end of the time of collection.

### Fatty acids, phospholipids, and choline analyses

A representative 1 ml aliquot of collected human milk was shipped on dry ice to the Nestle Research Center (Switzerland) for analyses of fatty acids (FAs) and phospholipids, whereas choline was analyzed at UNC-Chapel Hill. Specifically, direct quantifications of FAs were accomplished using gas chromatography as detailed in Cruz-Hernandez et al. (30), and the total monounsaturated fatty acids (TMUFA), total polyunsaturated fatty acids (TPUFA), TSFA, ARA, DHA, ARA-to-DHA ratio (ARA/DHA), and omega-6/omega-3 polyunsaturated fatty acids ratio (n-6/n-3) were obtained. In contrast, analyses of phospholipids were accomplished using high-performance liquid chromatography coupled with a mass spectrometer detector (31), which yielded phosphatidylcholine (PC), phosphatidylethanolamine (PE), phosphatidylinositol (PI), and sphingomyelin (SPH). Finally, quantification of choline and choline metabolites was performed using liquid chromatography–stable isotope dilution–multiple reaction monitoring/mass spectrometry (LC–SID–MRM/MS). Chromatographic separations were performed on an Acquity HILIC 1.6 μm 2.1 × 50 mm column (Waters Corp., Milford, CT, United States) using a Waters ACQUITY UPLC system, and free choline, phosphocholine (PCho), and glycerophosphocholine (GPC) were obtained.

### Assessments of cognition and temperament

Two measures, namely, the Mullen Scales of Early Learning (MSEL) and Infant Behavior Questionnaires—Revised (IBQ-R) (32), were employed to assess cognition and temperament, respectively. The MSEL includes five subscales, namely, fine motor (F.M.), gross motor (G.M.), visual reception (V.R.), receptive language (R.L.), and expressive language (E.L.). An early learning composite (E.L.C) score considered as the developmental quotient of infants was calculated by summing the T-scores of all subdomains excluding G.M. The MSEL was administered by trained staff at every visit. In contrast, the IBQ-R is a widely used 14-scale parent report measure designed to assess infant temperament. To reduce the number of variables, three factors were extracted by using three linear weighted averages of the 14 subscales. Specifically, we incorporated the weights of three latent factors reported in the exploratory factor analysis results of Gartstein and Rothbart (32) and obtained three personality traits, namely, surgency/extraversion (SUR), negative affectivity (NEG), and orienting/regulation (REG). The subscale items included in the above three factors and loading scales are provided in Supplementary Table 1. In our study, the MSEL or IBQ-R assessments in the first 6 months were obtained within 30 days from the collection of human milk samples.

### Statistical modeling for associations analyses in the first 6 months of life

All human milk nutrient concentrations were first normalized with mean zero and unit variance. Marginal association analyses between each individual nutrient and each subdomain of MSEL and IBQ-R scores were carried out by linear regression of each subdomain score on each nutrient. As nutrients could vary with postpartum duration, to ensure that age does not contribute to our analyses, age was included as a controlled variable. Other confounding factors including sex, data collection site, and household income (if < 75k) were also controlled in the above linear model, and the standardized regression coefficient was obtained between each nutrient and each MSEL/IBQ-R score. Household incomes of two infants were missing and imputed with the overall average. Specifically, we used the linear model $M\,0:\mathrm{MSEL}\,\mathrm{or}\,\mathrm{IBQ}∼\,1+\sum_{\text{k}=1}^{4}C_{\text{k}}+X_{j},j=1,\ldots ,\,p,$ where *C*_*k*_, *k* = 1,…, 4, are the four controlled confounders for each nutrient *X_j_*.

To evaluate the potential combined and conditional effects of human milk nutrients in association with MSEL and IBQ-R and to deal with the collinearity among the human milk nutrients, the correlation matrix among all nutrients was calculated. Subsequently, nutrients were clustered into several subgroups based on their correlation using the single linkage clustering analysis (SLCA) (33). The SLCA clustering, also known as the nearest-neighbor clustering, is one of the widely used approaches of hierarchical clustering. The SLCA approach was chosen as it offers intuitive interpretations of the results by using the minimum spanning tree; all pairs of nutrients exhibiting correlation coefficients greater than a predefined threshold *T*_*c*_ were combined into one sub-group. The optimal number of clusters was determined by maximizing the Dunn index (34). A higher Dunn’s index indicates a better clustering with either an increased minimal intercluster distance or a decreased maximal cluster diameter. Furthermore, in order to minimize redundancy and collinearity (35), the best subset selection model (36–38) was employed for nutrient selection. To reduce multi-collinearity, we only consider possible combinations from those with at most a single nutrient from each cluster. To formularize the model, we denote 𝒯 as the set of all the nutrients, and 𝒯_*k*_, *k* = 1,…,*p*, as p clusters obtained based on the SLCA clustering approach. The nutrient selection model is $M\,1:\mathrm{MSEL}\,\mathrm{or}\,\mathrm{IBQ}∼\,1+\sum_{k=1}^{4}C_{k}+\sum_{j=1}^{q}X_{j},X_{j}\in {𝒯}_{k_{j}}$, where *q* is the number of selected nutrients with  0 ≤ *q* ≤ *p* and *X*_*j*_ is the selected nutrient from the *k*_*j*_th cluster 𝒯_*k*_*j*__ with *k*_*j*_ ∈ {1,…,*p*} and *k*_*j*_ ≠ *k*_*l*_ if *k* ≠ *l*. Different combinations of nutrients *X*_*j*_ were fed into model *M*1, and the final selected nutrients will be determined by maximizing the adjusted *R*-squared of model *M*1 to reduce overfitting. The final selected nutrients were then used as the covariates and MSEL or IBQ-R obtained within 30 days before or after the collection of human milk samples as the outcomes for the regression model to uncover the potential associations. The overall significance was reported using the ANOVA F-statistics in comparison with the reduced baseline model $M\,2:\mathrm{MSEL}\,\mathrm{or}\,\mathrm{IBQ}∼\,1+\sum_{\text{k}=1}^{\text{q}}C_{\text{k}}$. The FDR adjustment was adopted for controlling type I errors of multiple comparisons. To assess whether the identified nutrients predict cognitive and temperament scores of new subjects, multiple regression models with the above-selected nutrients were evaluated by squared cross-validation (CV) errors and prediction correlations through 100 repetitions of 5-fold CV.

### Model evaluation on follow-up visits beyond 6 months

To evaluate whether the identified associations of human milk nutrients and cognition/temperament persisted beyond the first 6 months of life in the same subjects, the subjects with follow-up visits were binned into three age groups: 6–9 months, 9–12 months, and 12–18 months. Each observation within each age group corresponded to a unique subject. The regression models trained above were used to predict MSEL and IBQ-R scores (predicted scores). The correlation and *p*-value based on Pearson’s *t*-test between the predicted and observed scores were evaluated.

## Results

**FIGURE 1:**
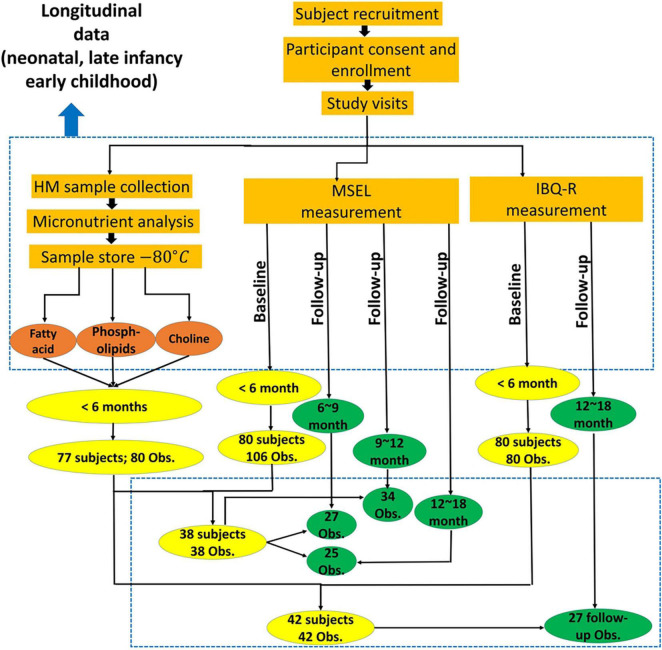
Study flowchart provides the overall experimental details.

Of the 54 subjects, cognition was assessed using MSEL in 38 subjects (4.64 ± 0.89 months; 12 male subjects), whereas temperament was assessed using IBQ-R in 42 subjects (4.48 ± 0.73 months; 16 male subjects) and both were available in 26 subjects (4.81 ± 0.71 months; 9 male subjects). After the initial assessments, the follow-up assessments of MSEL were available in 27, 34, and 25 subjects at 6–9 (7.63 ± 0.87 months; 9 male subjects), 9–12 (10.5 ± 0.97 months; 10 male subjects), and 12–18 months old (14.02 ± 1.08 months; 8 male subjects), respectively. In contrast, IBQ-R was obtained from 7, 7, and 27 subjects between 6 and 9 (mean: 243.9 ± 12.3 days), 9 and 12 (mean: 341.1 ± 18.8 days), and 12 and 18 (mean: 13.34 ± 0.79 months) months, respectively (Figure 1 and Table 1). Detailed demographic information on infants and their mothers including infant age, sex, anthropometrics, measured breast milk gross composition such as fat, carbohydrates, proteins, and energy, as well as household income and mother’s education, is summarized in Table 1.

**TABLE 1:** Characteristics of the participants.^a^

	All subjects	MSEL^b^ (<6 m)	MSEL (6–9 m)	MSEL (9–12 m)	MSEL (12–18 m)	IBQ-R^c^ (<6 m)	IBQ-R (12–18 m)
Numbers of subjects	54	38	27	34	25	42	27
Sex (male)	19	12	9	10	8	16	4
Age (months)	4.43 (0.83)	4.64 (0.89)	7.63 (0.87)	10.5 (0.97)	14.02 (1.08)	4.48 (0.73)	13.34 (0.79)
Birth length (cm)	52.35 (2.50)	52.29 (2.35)	51.85 (2.18)	52.26 (2.49)	52.27 (2.20)	52.32 (2.45)	52.51 (2.47)
Gestation ages (days)	280.6 (6.0)^d^	279.3 (6.2)	279.1 (5.5)	279.3 (6.2)	278.4 (6.5)	280.2 (5.9)	280.8 (5.3)
Birth weight (g)	3630 (420)	3548 (399)	3469 (396)	3534 (419)	3523 (415)	3619 (428)	3611 (388)
Weight at visit (g)	6818 (1081)	6872 (1244)	8649 (1658)	9396 (1379)	10115 (1160)	6936 (824)	1028 (1284)
Height at visit (cm)	63.81 (4.54)	64.14 (5.15)	68.47 (6.10)	72.98 (4.70)	75.90 (6.64)	64.62 (4.27)	76.38 (8.24)
Household incomes (n)							
<75k	18	13	7	12	8	14	9
75k–150k	28	18	15	17	13	23	16
150k<	6	5	4	3	3	5	2
Not answered	2	2	1	2	1	0	0
Maternal education							
≥Graduate^e^	26	15	9	14	12	17	23
<Graduate	27	22	18	19	13	10	19
Unavailable	1	1	0	1	0	0	0

^a^Provided are the means with standard deviations in parentheses.

^b^MSEL, Subjects with Mullen Scales of Early Learning scores taken.

^c^IBQ-R, Subjects with Infant Behavior Questionnaires—Revised scores taken.

^d^The gestation age range for all subjects is 268–292 days.

^e^≥Graduate: subjects with a graduate degree; < Graduate: subjects without a graduate degree.

**FIGURE 2:**
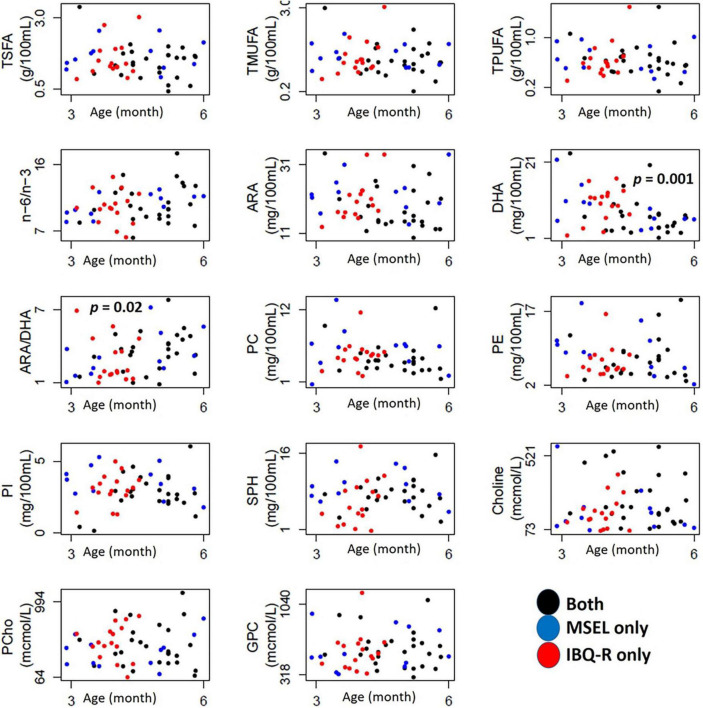
Scatterplots of the 14 nutrients with age in months (x-axis) for subjects assessed with MSEL (blue circles), IBQ-R (red circles), and both (black circles). TMUFA, total monounsaturated fatty acids; TPUFA, polyunsaturated fatty acid; TSFA, saturated fatty acid; ARA, arachidonic acid; DHA, docosahexaenoic acid; ARA/DHA, the ARA-to-DHA ratio; n-6/n-3, omega-6/omega-3 polyunsaturated fatty acids ratio; PC, phospholipids; PE, phosphatidylethanolamine; PI, phosphatidylinositol; SPH, sphingomyelin; Choline, free choline; PCho, phosphocholine; GPC, glycerophosphocholine; SUR, surgency; NEG, negative affectivity; REG, regulation; mcmol, micromoles.

Figure 2 shows how the 14 human milk nutrients vary with age during the first six postpartum months, while the summary statistics of their individual concentrations are provided in Table 2. Among these nutrients, DHA decreases (*p* < 0.002), while ARA/DHA ratio (*p* < 0.03) increases with postpartum age. As some of the subjects only had MSEL, but not IBQ-R, or *vice versa* during the first 6 months of life, the mean values of each nutrient from the MSEL dataset and the IBQ-R dataset were compared using the two-sided two-sample *t*-test. No significant differences were observed, suggesting that there is no selection bias in the mean nutrient concentrations. In addition, the MSEL and IBQ-R scores are provided in Supplementary Tables 2, 3, respectively, which are within the normal ranges of both scales.

**TABLE 2:** Composition of fatty acids, phospholipids, and choline metabolites in human milk in the first six postpartum months.

Nutrients	Mean (SD)	Median	(Lower) 5%	(Upper) 95%
TSFA (g/100ml)	1.54 (0.59)	1.43	0.76	2.64
TMUFA (g/100ml)	1.32 (0.53)	1.21	0.69	2.19
TPUFA (g/100ml)	0.64 (0.26)	0.6	0.33	1.03
n-6/n-3	10.57 (2.27)	10.14	7.39	14.37
ARA (mg/100ml)	20.07 (6.32)	19.15	12.21	33.99
DHA (mg/100ml)	8.35 (5.14)	7.09	2.58	18.14
ARA (% of total fatty acids)	0.60 (0.15)	0.60	0.40	0.80
DHA (% of total fatty acids)	0.24 (0.14)	0.19	0.11	0.52
ARA/DHA	3.24 (1.73)	3.2	1.01	6.15
PC (mg/100ml)	4.99 (2.58)	4.6	1.93	10.51
PE (mg/100ml)	7.53 (3.98)	6.4	3.45	16.39
PI (mg/100ml)	3.06 (1.19)	2.94	1.24	5
SPH (mg/100ml)	7.7 (3.61)	7.66	1.86	14.19
Choline (mcmol/l)	216.49 (140.47)	173.5	72.5	530.45
PCho (mcmol/l)	448.85 (235.64)	439.56	120.85	834.8
GPC (mcmol/l)	568.53 (192.07)	528.62	335.08	931.9

While the focus of our study was to jointly analyze a wide array of human milk nutrients in association with cognition and temperament in typically developing children, the marginal associations of each nutrient with MSEL and IBQ-R scores are given in Tables 3, 4, respectively. A significant association between ARA and SUR (*r* = 0.54, *p* = 0.0006, adjusted *p* = 0.024, 95% CI = [0.25, 0.83]) was observed. Several other nutrients including TSFA (*r* = 0.35, *p* = 0.035, 95% CI = [0.03, 0.68]), TPUFA (*r* = 0.35, *p* = 0.031, 95% CI = [0.03, 0.67]), and PCho (*r* = 0.46, *p* = 0.003, 95% CI = [0.17, 0.76]) were positively associated with SUR; TPUFA (*r* = 0.32, *p* = 0.046, 95% CI = [0.007, 0.64]) and ARA (*r* = 0.39, *p* = 0.015, 95% CI = [0.08, 0.70]) were associated with REG. In addition, marginal negative associations between G.M. and TSFA (the standardized regression coefficient *r* = −0.40, *p* = 0.01, and 95% confidence interval (CI) = [−0.71, −0.08]) and TMUFA (*r* = −0.38, *p* = 0.02 and 95% CI = [−0.70, −0.05]) and a positive association between DHA and R.L. (*r* = 0.38, *p* = 0.049 and 95% CI = [0.002, 0.76]) were observed, although all of these associations did not pass the FDR control due to limited sample size.

**TABLE 3:** Standardized regression coefficients of the human milk nutrients with the Mullen Scales of Early Learning scores.^a^

Nutrients	Composite score	Gross motor	Visual reception	Fine motor	Receptive language	Expressive language
TSFA	−0.25	−***0.40 (0.01)***^b^	−0.07	−0.21	−0.24	−0.19
TMUFA	−0.15	−***0.38 (0.02)***	−0.07	−0.15	–0.12	−0.08
TPUFA	−0.22	−0.27	−0.18	−0.11	−0.19	−0.09
n-6/n-3	0.10	0.14	0.23	−0.06	0.02	0.05
ARA	−0.06	−0.25	−0.08	0.00	−0.11	0.06
DHA	0.25	−0.08	0.02	0.14	***0.38 (0.049)***	0.07
ARA/DHA	−0.13	0.20	−0.14	−0.15	−0.09	0.06
PC	0.05	−0.28	−0.03	−0.11	0.22	−0.03
PE	0.04	−0.22	−0.14	−0.26	0.17	0.26
PI	−0.16	−0.19	−0.05	−0.21	−0.13	−0.11
SPH	−0.30	−0.26	−0.37	−0.31	−0.13	−0.10
Choline	0.03	0.09	0.03	−0.07	−0.10	0.29
PCho	−0.02	−0.03	0.03	−0.23	0.20	−0.09
GPC	−0.05	0.07	0.21	−0.10	−0.23	0.06

^a^Confounding factors: age, sex, site, and household income (if < 75k) were controlled.

^b^Statistics in bold italics are standardized coefficients with raw *p*-values (in parentheses) of < 0.05.

**TABLE 4:** Standardized regression coefficients of the human milk nutrients with the Infant Behavior Questionnaires—Revised factors.^a^

Nutrients	Surgency	Negative affectivity	Regulation
TSFA	***0.35 (0.035)*** ^b^	0.24	0.09
TMUFA	0.28	0.19	0.28
TPUFA	***0.35 (0.031)***	0.18	***0.32 (0.046)***
n-6/n-3	0.00	−0.12	0.06
ARA	***0.54 (0.0006)*** *	0.30	***0.39 (0.015)***
DHA	0.15	0.24	0.12
ARA/DHA	0.02	−0.12	−0.07
PC	0.24	0.17	0.23
PE	0.38	0.23	0.23
PI	0.26	0.23	0.09
SPH	0.09	0.12	0.07
Choline	−0.19	−0.13	−0.07
PCho	***0.46 (0.003)***	0.05	0.18
GPC	0.09	0.22	0.11

^a^Confounding factors: age, sex, site, and household income (if < 75k) were controlled.

^b^Statistics in bold italics are standardized coefficients with raw *p*-values (in parentheses) of < 0.05.

*Adjusted *p* < 0.05 after FDR correction.

**FIGURE 3:**
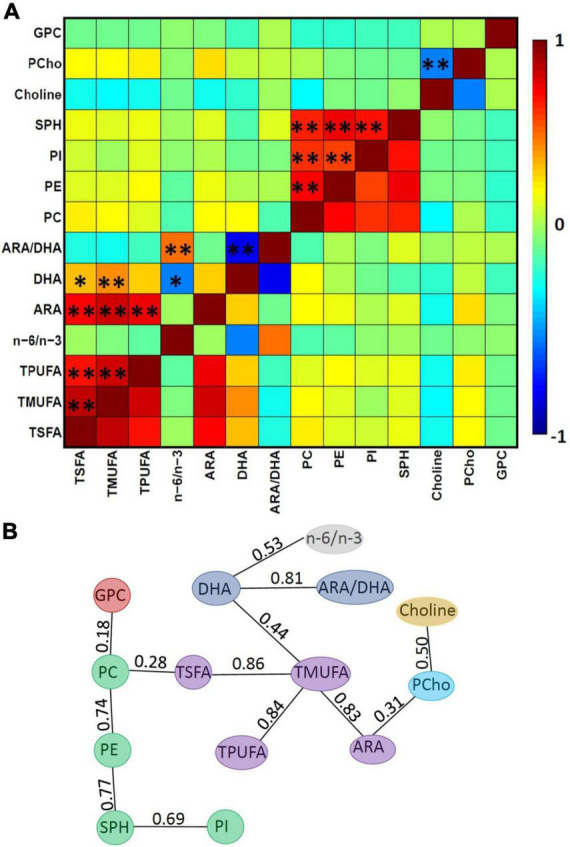
Relations between nutrients. **(A)** Pearson’s correlation between each pair of the 14 nutrients, where single asterisks (*) and double asterisks (**) represent a raw *p*-value of <0.05 and adjusted *p* < 0.05, respectively, using the standard Pearson correlation test and the FDR correction. **(B)** The minimum spanning tree, where colors represent different clusters and numbers represent the correlation coefficients between a given pair of nutrients. Note the optimal clustering result is stable for 0.53 < T_*c*_ ≤ 0.69. In addition, the minimum spanning tree shows that the strongest correlation between the clusters (PC, PI, PE, and SPH) and (TSFA, TMUPA, TPUFA, and ARA) is between the pair of TSFA and PC (0.28). It implies that only when T_*c*_ ≤ 0.28, these two clusters will merge together, while PCho and choline are closer in the sense that these two will merge when T_*c*_ ≤ 0.50.

We further tested whether human milk nutrients are correlated. Evidently, many of the human milk nutrients were highly correlated (Figure 3A). In particular, the nutrients in the phospholipid family were highly correlated. As outlined above, to minimize collinearity, the single linkage clustering analysis was used to cluster highly correlated nutrients into one group and the Dunn index was used to determine the optimal number of clustered nutrient groups. The rationale here is to keep highly correlated nutrients in the same group while splitting those less correlated nutrients into different groups. The optimal number of clusters was 7, which exhibited the largest Dunn index (Table 5). Note that the optimal clustering result was stable for 0.53 < *T*_*c*_ ≤ 0.69. In addition, the memberships of groups 1–3 were stable independent of the *T*_*c*_ for 0.53 < *T*_*c*_ ≤ 0.77. The minimum spanning tree representing the smallest sum of distances to touch all vertices of the graph of the optimal clustering results is shown in Figure 3B.

**TABLE 5:** Correlation and grouping of the 14 nutrients.

	Clustering result 1	Clustering result 2	Clustering result 3
	
	0.53 < T_*c*_^a^ ≤ 0.69	0.69 < T_*c*_ ≤ 0.74	0.74 < T_*c*_ ≤ 0.77
Group 1	TSFA, TMUFA, TPUFA, ARA	TSFA, TMUFA, TPUFA, ARA	TSFA, TMUFA, TPUFA, ARA
Group 2	n-6/n-3	n-6/n-3	n-6/n-3
Group 3	DHA, ARA/DHA	DHA, ARA/DHA	DHA, ARA/DHA
Group 4	PC,PE,PI,SPH	PC, PE, SPH	PE, SPH
Group 5	GPC	Choline	Choline
Group 6	PCho	PCho	PCho
Group 7	Choline	GPC	GPC
Group 8	-	PI	PC
Group 9	-	-	PI
Dunn index	0.64	0.44	0.41

^a^T_c_, The threshold values for the correlation coefficients.

**FIGURE 4:**
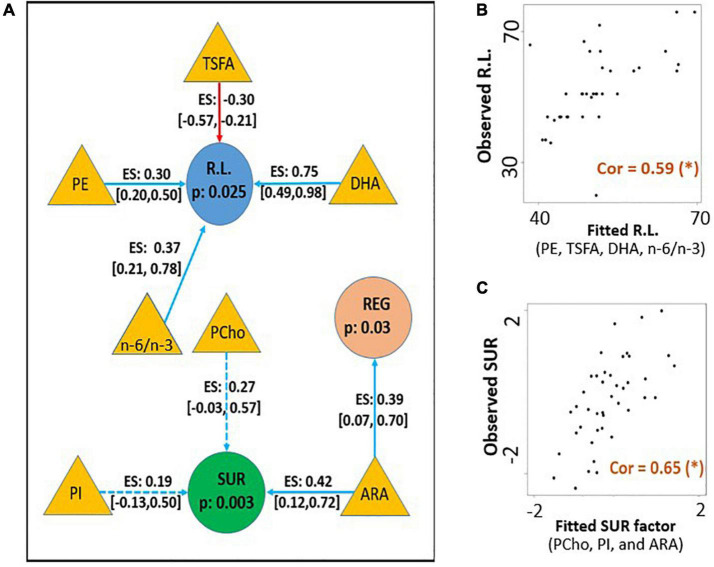
**(A)** The identified conditional effects of human milk nutrients (filled triangles) on receptive language score (R.L., filled blue circles) and surgency (SUR, filled green circles) using linear regression. The blue and red arrows represent the positive and negative associations, respectively. The corresponding effect size (ES), confidence interval of coefficients for each association, and FDR-corrected *p*-values for the regression model are provided. Dotted lines indicate the regression coefficient of the covariate is not significant, but the inclusion of the corresponding nutrient improves model fitting with a higher adjusted *R*^2^ value. **(B)** The association between the experimentally obtained R.L. and the combined effect of selected human milk nutrients DHA, n-6/n-3, PC, and TSFA (fitted R.L.). **(C)** The association between the experimentally obtained SUR and the combined effect of selected human milk nutrients ARA, PCho, and PI (fitted SUR). Pearson’s correlation between the fitted and observed measurements is included in panels **(B,C)** with (*) indicating adjusted *p* < 0.05.

**FIGURE 5:**
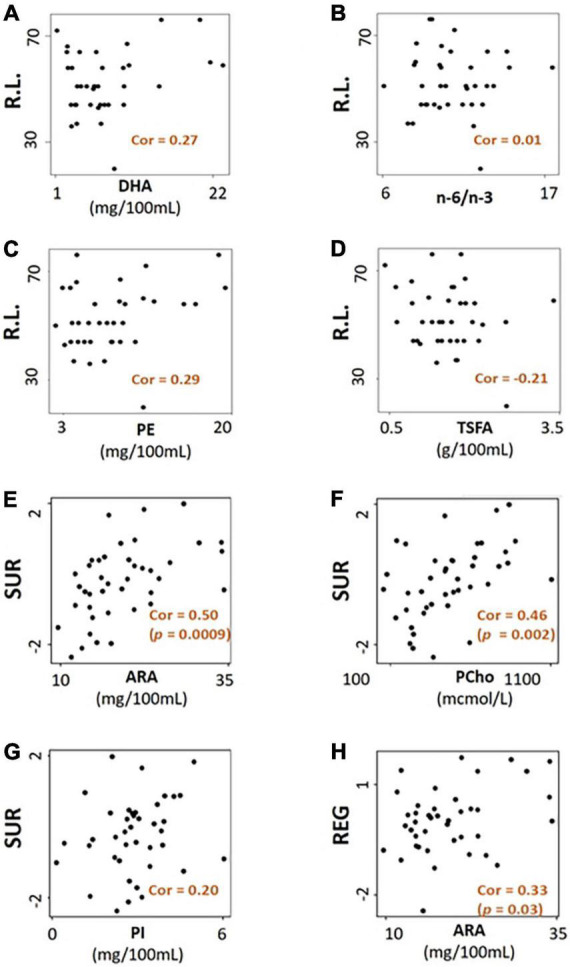
The scatterplots of selected nutrients for receptive language score (R.L.) are shown in panels **(A–D)**, surgency (SUR) in panels **(E–G)**, and regulation (REG) in panel **(H)** mcmol, micromoles.

**FIGURE 6:**
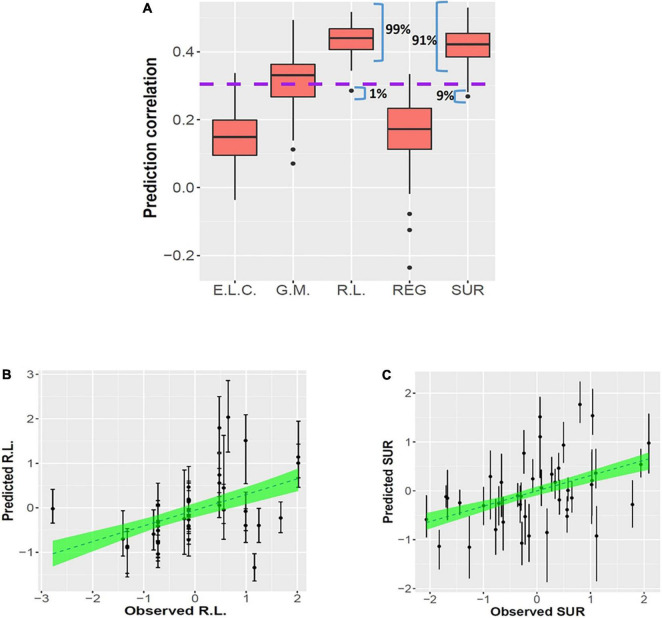
Prediction results for linear regression models from 100 random splits of 5-fold CV. Panel **(A)** shows the boxplots of 100 Pearson’s correlations between the observed and predicted MSEL scores, including the early learning composite score (E.L.C.), gross motor (G.M.), and receptive language score (R.L.) and IBQ-R factors including the regulation (REG) and surgency (SUR), respectively, based on 100 repetitions of CVs. The purple dashed line represents the significance threshold of the correlation at α = 0.05 with *n* = 38 subjects. Panels **(B,C)** show the observed R.L. (left) and SUR (right) vs. the predicted R.L. and SUR of the 100 times’ predictions, respectively. The dashed lines and the green areas show the mean and the 95% confidence intervals of the fitted linear slopes between the observed and predicted observations, respectively.

Significant combined associations of human milk nutrients were observed for R.L. (MSEL) and SUR (IBQ-R) summarized in Figure 4A. Specifically, for receptive language, the final linear regression model includes DHA, n-6/n-3 ratio, PE and TSFA (-), and *R*-squared = 0.39 (adjusted *p* = 0.025), where the information provided in the parentheses indicates the sign of the coefficients and positive otherwise. In contrast, for SUR, the model includes ARA, PI, and PCho with *R*-squared = 0.43 and adjusted *p* = 0.003. Finally, a positive association between ARA and REG with *R*-squared = 0.23 and adjusted *p* = 0.03 was observed. The scatterplots of the combined associations between the experimentally obtained and fitted R.L. and SUR using the identified association model are shown in Figures 4B,C, respectively. More detailed coefficients, confidence intervals, and *p*-values for each model are given in Table 6. In addition, the scatterplots of the associations between each of the selected nutrients for R.L., SUR, and REG are shown in Figure 5. Finally, a 5-fold CV between the observed and predicted MSEL and IBQ-R scores was conducted. The boxplots of Pearson’s correlations between the observed and predicted MSEL and IBQ-R scores from 100 random splits of 5-fold CV are shown in Figure 6A. The boxplots imply that in 99% and 91% cases of the 100 random splits of CV repetitions, the correlation between the observed and predicted R.L. scores and SUR was significant, respectively, whereas in most cases the association with the observed REG was not significant. These results showed a stronger prediction power of R.L. and SUR, but not REG. The observed (X-axis) versus the predicted R.L. (Figure 6B) and SUR (Figure 6C) of the 100 times’ predictions are also provided. The mean and the 95% confidence interval of prediction correlations (based on the random splits in CV) were 0.434 and [0.349, 0.501] for R.L. and 0.409 and [0.281, 0.509] for SUR, respectively. These results showed model robustness of predictions against training data perturbations.

**TABLE 6:** Multiple regression results for MSEL scores/IBQ-R factors with the selected nutrients.

	Composite score	Gross motor	Receptive language	Surgency	Regulation
*R*-squared	0.31	0.42	0.36	0.43	0.23
Adjusted *R*-squared	0.12	0.26	0.18	0.31	0.12
Raw *p*-value^a^	0.05	0.05	0.005	0.0008	0.017
Adjusted *p*-value^b^,^c^	0.05	0.11	**0.025**	**0.003**	**0.03**
TSFA^d^,^e^,^f^	−0.42 (0.03)	-	−0.30 (0.05)	-	-
TPUFA	-	-	-	-	-
TMUFA	-	−0.51	-	-	-
ARA	-	-	-	0.42 (0.008)	0.39 (0.02)
n-6/n-3	0.26 (0.19)	0.19	0.37 (0.006)	-	-
DHA	0.55 (0.02)	0.30	0.75 (0.002)	-	-
ARA/DHA	-	-	-	-	-
PC	-	-	-	-	-
PE	-	-	0.30 (0.07)	-	-
PI	-	-	-	0.19 (0.24)	-
SPH	−0.22 (0.23)	−0.24	-	-	-
Choline	-	-	-	-	-
PCho	-	-	-	0.27 (0.08)	-
GPC	-	-	-	-	-
Age	−0.006 (0.98)	0.28 (0.12)	0.30 (0.03)	0.18 (0.26)	0.18 (0.31)
Sex (if Male)	0.03 (0.87)	0.08 (0.61)	−0.01 (0.79)	−0.03 (0.83)	−0.004 (0.98)
Site (if UNC)	0.20 (0.29)	0.44 (0.01)	0.10 (0.63)	−0.32 (0.05)	0.22 (0.21)
Income (if < 75k)	−0.13 (0.44)	0.14 (0.35)	−0.15 (0.21)	−0.15 (0.34)	0.06 (0.71)

^a^This row shows ANOVA F-test *p*-values comparing the full and reduced models (confounders only); the results with raw *p*-values of > 0.05 are not shown.

^b^*p*-values in this line indicate the *p*-values after FDR correction for multiple comparisons.

^c^Adjusted *p*-values less than 0.05 are shown in bold.

^d^Numbers in parentheses show the *p*-values of the coefficients using the t-test statistic.

^e^Hyphen “-” indicates that this variable is not selected in this model based on the best subset selection feature selection algorithm.

^f^Coefficients here are standardized linear regression coefficients.

**FIGURE 7:**
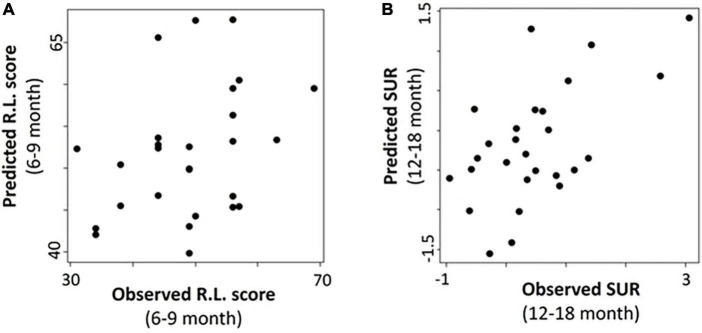
Association of the predicted and observed receptive language score (R.L.) and surgency (SUR) at the follow-up ages. **(A)** Predicted vs. observed R.L. at 6–9 months. **(B)** Predicted vs. observed SUR at 12–18 months.

Finally, we performed exploratory analyses for predicting the two outcome measures at the follow-up observations using results obtained < 6 months old. With the limited sample sizes for IBQ-R at the 6–9- and 9–12-month follow-up visits (Table 1 and Figure 1), the IBQ-R analyses were only performed for the 12–18-month age group, whereas, MSEL was conducted for all three age bins. The correlation coefficients (*p*-values) for MSEL were 0.35 (*p* = 0.07), 0.14 (*p = 0.43*), and −0.21 (*p = 0.32*) at 6–9, 9–12, and 12–18 months, respectively; the correlation coefficient (*p*-value) for SUR at 12–18 months was 0.58 (*p = 0.002*). Evidently, the associations between the predicted and observed R.L. decrease with age with the best performance at 6–9 months (*p = 0.07*; Figure 7A). In contrast, a significant association between the predicted and observed SUR at 12–18 months was observed (*p* = 0.002; Figure 7B).

## Discussion

The human milk produced by each mother for her infant has a unique composition and contains a myriad of different lipids, vitamins, minerals, bioactive carbohydrates, proteins, and immune factors that evolve in tandem with the growth and developmental needs of the infant (39). In addition, maternal diet and lifestyle can also affect human milk composition, especially the lipid components (40). While several studies have shown positive associations of human milk bioactive components and nutrients with brain development (29, 41), most of them have largely considered one or only few nutrients. Christian et al. (27) recently pointed out that these approaches may be overly simplistic and failed to consider the complex synergetic effects of human milk nutrients on infant development and health. They advocated the need for a paradigm shift by considering human milk nutrients as a biological system in future studies aiming to discern the benefits of human milk nutrients. To this end, we collected human milk samples, concurrently assessed the cognition and temperament of BF infants, utilized advanced statistical models to jointly consider 14 widely evaluated human milk nutrients spanning the three families, namely, choline, fatty acids, and phospholipids, and reported combined associations between these nutrients and cognition/temperament during the first 6 months of life. Furthermore, whether the effects of identified nutrients during the first 6 months persist through to 1.5 years old was also evaluated.

An analysis of the temporal trends in human milk nutrients over the first six postnatal months revealed that DHA exhibited a significant negative age effect and choline remained relatively stable, consistent with those reported previously (42). In contrast, while the phospholipids were stable with age, several studies have reported an increase in lactation (42, 43). Human milk DHA content was highly variable (ranging from 0.08% to 0.71%) with a median concentration of 0.19%, which is below the worldwide average (0.37%) and those reported from Southeast Asian regions (42, 44). Nevertheless, lower levels of human milk DHA have been reported in the North American region (45), possibly due to low habitual intake of seafood in the inland areas, and those levels are strongly impacted by maternal DHA intake (46). In addition, the median human milk ARA of 0.60% in our cohort was higher compared with the global average (0.55 %), leading to a higher ARA/DHA ratio (3.24) when compared with that reported in the literature (1.5–2) (47). The human milk n-6/n-3 fatty acid ratio in our study (mean 10.57), while comparing some geographies, was higher than others (48), suggesting differences in either body fat composition and mobilization of fat stores or dietary habits such as consumption of LA-rich vegetable oils (49). The levels of most PLs (PC and PE) in our study were comparable to those reported from Singapore (42). We observed higher concentrations of free choline and GPC, but lower levels of PCho, compared with reports from Canada and Cambodia (50), possibly due to differences in dietary choline intake and amounts of choline available from the maternal circulation. Taken together and compared with other studies, our findings highlight that several human milk nutrients in the choline, fatty acids, and phospholipids families may show geographical differences across regions reflecting differences in diet and lifestyle.

The potential interplay between nutrients and early brain development has been widely recognized (51). Our brains undergo rapid development during the first years of life by establishing new synapses (synaptogenesis), removing excessive synapses (pruning), myelination (52), and forming highly complex yet efficient brain functional networks enabling the performance of cognitive tasks and social behaviors (53). During these dynamic and highly energy-demanding brain developmental processes, an appropriate nutrient supply is key to healthy neurodevelopment in infancy. Our results show that while the individual content of PE, DHA, n-6/n-3, or TSFA does not show a significant association on its own, a combined effect is observed on receptive language (R.L.). In contrast, ARA by itself is significantly associated with SUR (adjusted *p* = 0.024), whereas neither PCho nor PI is associated. Nevertheless, three nutrients, namely, ARA, PCho, and PI, jointly exhibit an improved association and predictive performance with SUR in terms of elevated model significance, decreased AIC, and CV errors. Importantly, we also observed a significant association between ARA and REG. This finding differs from the marginal association analyses where no associations were observed between REG and ARA after FDR correction. Largely, this finding is not surprising. Specifically, FDR correction was employed for the marginal associations to control for 14 nutrients and subscales of scores. In contrast, as multiple regression was employed with features selected using approaches outlined above, the FDR correction was only employed to control for subscales of scores, but not for 14 nutrients. Instead, 5-fold cross-validation was used to evaluate the possibility of overfitting. Indeed, CV results showed that the identified association between (PE, DHA, n-6/n-3, TSFA) and R.L. and (ARA, PCho, and PI) and SUR was robust, but not between ARA and REG. Therefore, caution should be taken when interpreting the association between ARA and REG. Future studies with a larger sample size are warranted to further evaluate the association between ARA and REG.

Several key findings of our study deserve additional discussion. First, while MSEL assesses five domains of cognition, significant joint associations were only observed between PE, DHA, n-6/n-3, TSFA, and receptive language in our study. Several studies have previously evaluated the potential associations between individual human milk nutrients and infants’ language ability. However, thus far the results are inconsistent and sometimes controversial. Although the effect of PE on language abilities has not been specifically reported in the literature, the milk fat globule membrane (MFGM), which is a diverse mixture of bioactive components including phospholipids, has been shown to improve visual function, language, and motor domains in both term and preterm infants (16, 54, 55). In contrast, DHA has been reported to improve language ability. However, a recent comprehensive review by Gawlik et al. (56) has concluded that the current evidence of DHA supplementation on language development is limited and non-conclusive. Ramos et al. (57) reported that a higher ratio of the linoleic (n-6 fatty acid) to the alpha-linolenic acid (n-3 fatty acid) could exert beneficial effects for R.L. in human milk-fed preterm infants. Nevertheless, their results are contrary to the body of literature showing that n-6/n-3 ratio in the maternal blood and diet is negatively associated with the vocabulary and verbal fluency of their infants (58, 59). Although the use of the n-6/n-3 ratio to assess potential health benefits is debatable due to the observed positive correlation between ARA and DHA concentrations in maternal plasma phospholipids (60–62), an appropriate balance between the two has been shown as an important factor contributing to cognitive development during infancy (63, 64). Nonetheless, inconsistent results are reported in the literature. The reasons may be found in differences in methodology, infant ages at assessment, and length of supplementation among these studies. Alternatively, failure to consider possible joint effects of multiple nutrients could also contribute to the observed inconsistent results reported in the literature, underscoring the importance of considering the strong dependency between human milk nutrients and the need to jointly analyze multiple nutrients.

Second, the general consensus is that temperament traits emerge early in life and have a strong genetic and neurobiological basis (65). However, what is less understood is the role of BF and human milk nutrients in shaping these offspring behavioral traits. Our study highlights that human milk ARA (adjusted *p* = 0.024) alone (but not DHA) and the combined effects of ARA, PCho, and PI exhibited a significant association with SUR, which includes high-intensity pleasure, smiling and laugher, perceptual sensitivity, vocal reactivity, and activity. Tallima and Ridi reported that the downstream metabolites of ARA such as eicosanoids or endocannabinoids play a critical role in brain reward signaling, motivational processes, emotion, stress responses, and pain (26), which may further shape infants’ behavioral traits. Our results differ from the recent findings of Hahn-Holbrook et al. (25), who showed that higher n-3 PUFAs in human milk (more specifically ALA which is a precursor of DHA), but not any of the n-6 PUFAs, were associated with significantly less sadness and distress to limitations (25). It is plausible that differences in experimental design (varying ages among subjects in our cohort vs. all assessed at 3 months old by Hahn-Holbrook) and the limited sample size in our study may have contributed to the different findings. Nevertheless, compared with the human milk nutrients associated with receptive language and SUR in our study, it appears that there may be distinct LCPUFA roles (DHA vs. ARA) for temperament and language functions, respectively. This finding could be of importance particularly in light of the legislation adopted by the European Commission in 2016 stipulating that all infant and follow-up formulas marketed in Europe must contain DHA in the amount of 20–50 mg/100 kcal, but without any requirement for ARA starting 2020 (66). Our study highlights the importance of ARA, which by itself and jointly with PCho and PI exhibit a significant association with SUR. Nevertheless, future studies with a larger sample size are warranted to determine whether ARA or its metabolites have a distinct role from that of DHA on behavioral development.

Finally, our findings also reinforce the importance of choline (PCho) during the BF period in supporting the developmental needs of the infant (67). While no study has specifically looked at HM choline levels and infant behavior, phosphatidylcholine supplementation during pregnancy has shown to result in fewer attention problems and less social withdrawal at infant age of 40 months by increasing activation of the α7-nicotinic acetylcholine receptor (68), and newborns of mothers with higher choline levels during gestation (and concurrent infection) had improved inhibition of auditory cerebral response, while their infants at 1 year of age had improved development of self-regulation, approaching the level of children of mothers without infection at 1 year of age (69). Equally, phospholipid supplementation in early life (*via* milk fat globule membrane-supplemented formula) was shown to be associated with fewer parent-reported behavioral problems in their children and improved behavioral regulation (70).

The regression models elucidating potential relations between the observed human milk nutrients and cognition/temperament during the first 6 months of life were evaluated on subjects whose follow-up MSEL and IBQ-R were available at 6–18 months of age. Using association analyses, the correlation between the predicted and observed SUR scores was significant at 12–18 months of age (*r* = 0.58, *p* = 0.002), and there is a trend toward significance (*p* = 0.07) between the predicted and observed R.L. at 6–9 months. These results suggest that the MSEL or SUR scores beyond 6 months can be possibly predicted using the derived regression models encompassing the combined effects of human milk nutrients from the first 6 months of life. Although the limited sample sizes for SUR at 6–9 and 9–12 months made it difficult to determine whether the findings observed at 12–18 months are also present during the two age periods, our results appear to suggest that the effects of human milk on temperament persist longer than those for R.L. Specifically, the association of SUR is quite strong at 12–18 months, while the association of R.L. exhibits a trend toward significance at 6–9 months (*p* = 0.07) but continues decreasing at 9–12 and 12–18 months. It is, however, worth noting that other unobserved confounders could affect the future cognition and temperament, such as type of confounding factors (amount and variety), solid food intakes, and environmental stimulations. As a result, the effects of human milk nutrients that infants received < 6 months on cognition/temperament could diminish with age. Nevertheless, our results suggest that the SUR is less affected by unobserved confounders and thus may be more predictable from baseline. Future studies need to carry out an in-depth investigation to confirm our results.

It should be noted that our studies consisted of two main limitations. First, the limited sample size could lead to biases, including collinearity among the human milk nutrients, overfitting, and instability due to potential outliers. To this end, we carefully carried out several analysis steps to mitigate potential biases. To reduce the multi-collinearity caused by the limited sample size and potential correlations among the nutrients, we used a data-driven approach to classify human milk nutrients into six clusters. Nutrients in different clusters are significantly less correlated compared with those within the same cluster. By selecting only one nutrient from each cluster for the optimal model, we could minimize the collinearity. To reduce potential overfitting caused by the limited sample size, we evaluated the prediction performance using cross-validation. Our results indicated that the predicted and the true MSEL and IBQ-R measures on the test set were significantly correlated. Finally, our results showed that the prediction performance with random alterations between the training and test data based on 100 repetitions of cross-validation was stable and consistently significant, suggesting the robustness against outliers. Second, it was reported that the gestational length could influence DHA and ARA levels among the milk samples, especially for preterm infants (71). In our study, the potential effects of gestational lengths (280.6 ± 6.0 days) on DHA and ARA levels were not considered. Future studies including the effects of gestational lengths on DHA and ARA should be considered.

## Conclusion

The development of cognitive and behavioral functioning is complex and likely involves the interplay of social, psychological, and biological factors. Nevertheless, human milk nutrients such as LCPUFAs, choline, and phospholipids may be modifiable contributors to cognitive and behavioral development, especially during the early BF period. Our results provide evidence that specific nutrients in human milk may act together to support cognitive and behavioral traits. However, these findings warrant replication in larger cohorts of BF infants with longitudinal follow-up for more definitive behavioral phenotyping, by controlling maternal diet and lifestyle, by an in-depth understanding of the mechanisms by which human milk nutrients jointly affect developmental trajectories, and by careful examination of the synergistic effects, if any, of these nutrients on functional outcomes.

## Data availability statement

The original contributions presented in this study are included in the article/Supplementary material, and further inquiries can be directed to the corresponding author.

## Ethics statement

All study activities were approved by the Institutional Review Boards of the University of North Carolina at Chapel Hill, Chapel Hill, NC and University of Minnesota, Twin Cities, MN. Written informed consent to participate in this study was provided by the participants’ legal guardian/next of kin.

## Author contributions

BH, JE, and WL designed the research. KB, BH, HH, JE, and WL conducted the research. TL, TS, ZZ, SC, DW, and WL analyzed the data or performed the statistical analysis. TL, TS, and WL wrote the manuscript. All authors had primary responsibility for the final content and read and approved the final version of the manuscript.
