# Supplementary material for: Joint analyses of human milk fatty acids, phospholipids, and choline in association with cognition and temperament traits during the first 6 months of life
Source: Front Nutr. 2022 Aug 24;9:919769. doi: 10.3389/fnut.2022.919769 (PMC9449418; doi:10.3389/fnut.2022.919769)
Supplement: Supplementary file 1 [file Table_1.DOCX]

**Joint Analyses of Human Milk Nutrients in association with cognition and temperament traits during the first 6 months of life**

## Li et al.

## Online Supplementary Material

The loadings of the IBQ-R latent factors based on the original 14 IBQ-R scores are listed in **Supplementary Table 1** for references. The descriptive statistics of the MSEL scores and the IBQ-R measurements (including the original 14 IBQ-R scores and the 3 IBQ-R latent factors) are provided in **Supplementary Table** **2** and **Supplementary Table** **3**, respectively.

**Supplementary Table 1: IBQ-R three factor loadings (weights) and subscales included in each factor.**

| IBQ-R |  |  |  |
| --- | --- | --- | --- |
| Composite Scales | **Subdomain** | | **Loadings/weights** |
| *Negative affectivity* | high intensity pleasure | | -0.25 |
|  | sadness |  | 0.79 |
|  | distress to limitations | | 0.69 |
|  | fear |  | 0.31 |
|  | falling reactivity | | -0.56 |
| *Surgency/Extraversion* | approach |  | 0.74 |
|  | vocal reactivity | | 0.74 |
|  | high intensity pleasure | | 0.69 |
|  | smiling and laughter | | 0.55 |
|  | activity level | | 0.49 |
|  | perceptual sensitivity | | 0.45 |
|  | cuddliness | | -0.24 |
|  | soothability | | 0.27 |
| *Orienting/Regulation* | smiling and laughter | | 0.33 |
|  | activity level | | -0.21 |
|  | distress to limitations | | -0.28 |
|  | low intesnity pleasure | | 0.70 |
|  | cuddliness | | -0.56 |
|  | duration of orienting | | 0.43 |
|  | soothability | | 0.43 |

**Supplementary Table 2: Descriptive statistics of the MSEL scores at the first 6 months.**

|  | Mean |  | Median | Std. | (Lower) 5% | (Upper) 95% |
| --- | --- | --- | --- | --- | --- | --- |
| Early Learning Composite | 103.24 |  | 105 | 13.10 | 79.85 | 122.6 |
| Expressive Language | 55.34 |  | 55 | 8.25 | 42 | 68.15 |
| Fine Motor | 46.82 |  | 46 | 7.88 | 39.1 | 59.15 |
| Gross Motor | 49.42 |  | 49 | 8.2 | 36.7 | 61.6 |
| Receptive Language | 52.45 |  | 51 | 11.67 | 36.85 | 72.6 |
| Visual Reception | 51.66 |  | 53 | 10.02 | 33.85 | 64.75 |

**Supplementary Table 3: Descriptive statistics of the 14 IBQ-R scores and the 3 IBQ-R latent factors during the first 6 months of life.**

|  | Mean | Median | Std. | (Lower) 5% | (Upper) 95% |
| --- | --- | --- | --- | --- | --- |
| Activity level | 4.3 | 4.47 | 0.76 | 3.07 | 5.4 |
| Distress to limitations | 3.56 | 3.56 | 0.87 | 2.38 | 4.88 |
| Fear | 2.22 | 1.87 | 0.88 | 1.31 | 3.86 |
| Duration of orienting | 3.68 | 3.74 | 0.85 | 2.25 | 4.92 |
| Smiling and laughter | 4.64 | 4.58 | 1.12 | 3.11 | 6.5 |
| High intensity pleasure | 5.41 | 5.31 | 0.85 | 3.92 | 6.75 |
| Low intensity pleasure | 5.31 | 5.38 | 0.87 | 4.24 | 6.45 |
| Soothability | 4.9 | 4.88 | 0.73 | 3.81 | 5.99 |
| Falling reactivity | 4.87 | 4.96 | 0.86 | 3.54 | 6.18 |
| Cuddliness | 6 | 6 | 0.57 | 5.13 | 6.81 |
| Perceptual sensitivity | 3.45 | 3.41 | 1.12 | 2 | 5.49 |
| Sadness | 3.5 | 3.43 | 0.95 | 2 | 5.48 |
| Approach | 4.14 | 4.08 | 1.08 | 2.33 | 5.82 |
| Vocal reactivity | 4.24 | 4.21 | 0.92 | 3 | 5.72 |
| Surgency | -0.2 | -0.14 | 1.05 | -1.97 | 1.63 |
| Negative affectivity | 0.13 | 0.29 | 1.04 | -1.44 | 1.44 |
| Regulation | 0.06 | 0.06 | 0.89 | -1.18 | 1.61 |
